# Maternal Hypertriglyceridemia in Gestational Diabetes: A New Risk Factor?

**DOI:** 10.3390/nu16111577

**Published:** 2024-05-23

**Authors:** Francisca Marques Puga, Diana Borges Duarte, Vânia Benido Silva, Maria Teresa Pereira, Susana Garrido, Joana Vilaverde, Marta Sales Moreira, Fernando Pichel, Clara Pinto, Jorge Dores

**Affiliations:** 1 Serviço de Endocrinologia, Diabetes e Metabolismo, Unidade Local de Saúde de Santo António, 4050-366 Porto, Portugal; 2 Serviço de Endocrinologia, Unidade Local de Saúde de Braga, 4710-243 Braga, Portugal; 3 Serviço de Endocrinologia, Unidade Local de Saúde do Tâmega e Sousa, 4560-136 Penafiel, Portugal; 4 Serviço de Obstetrícia e Medicina Materno-Fetal, Unidade Local de Saúde de Santo António, 4050-366 Porto, Portugal; 5 Serviço de Nutrição, Unidade Local de Saúde de Santo António, 4050-366 Porto, Portugal

**Keywords:** lipid profile, triglycerides, gestational diabetes, maternal–fetal outcomes, large for gestational age

## Abstract

Elevated maternal triglycerides (TGs) have been associated with excessive fetal growth. However, the role of maternal lipid profile is less studied in gestational diabetes mellitus (GDM). We aimed to study the association between maternal lipid profile in the third trimester and the risk for large-for-gestational-age (LGA) newborns in women with GDM. We performed an observational and retrospective study of pregnant women with GDM who underwent a lipid profile measurement during the third trimester. We applied a logistic regression model to assess predictors of LGA. A total of 100 singleton pregnant women with GDM and third-trimester lipid profile evaluation were included. In the multivariate analysis, pre-pregnancy BMI (OR 1.19 (95% CI 1.03–1.38), *p* = 0.022) and hypertriglyceridemia (OR 7.60 (1.70–34.10), *p* = 0.008) were independently associated with LGA. Third-trimester hypertriglyceridemia was found to be a predictor of LGA among women with GDM, independently of glycemic control, BMI, and pregnancy weight gain. Further investigation is needed to confirm the role of TGs in excessive fetal growth in GDM pregnancies.

## 1. Introduction

Gestational diabetes mellitus (GDM) is a prevalent pregnancy disorder that increases the risk of adverse outcomes for both the mother and the fetus [1]. Hyperglycemia and elevated maternal weight are well-known risk factors for increased fetal growth and the birth of large-for-gestational age (LGA) newborns [2]. Maternal hyperglycemia is related with an increased transfer of glucose across the placenta, resulting in fetal hyperglycemia [3]. This evokes an exaggerated fetal response with insulin and, ultimately, increased fetal growth [3].

Due to the fetus’s limited ability for de novo lipogenesis and fatty acid oxidation, it relies on maternal triglycerides for growth and development [4]. During pregnancy, several adaptative mechanisms occur, including a decreased lipoprotein lipase activity induced by an increased maternal insulin resistance, which leads to a 2–3-fold increase in plasma triglycerides (TGs) [4]. Maternal triglycerides, in the form of very-low density lipoprotein (VLDL) and chylomicrons, are then hydrolyzed to free fatty acids by the placental lipases and transferred to the fetal circulation [4].

Elevated maternal TG levels have been linked to pregnancy complications, including pre-eclampsia and excessive fetal growth [4,5,6,7]. Studies in GDM-complicated pregnancies with simultaneous evaluation of glycemic control are still scarce.

Our purpose was to increase our understanding of the potential role of lipid metabolism disturbances in LGA newborns in pregnancies complicated with GDM. The primary aim of this study was to assess the role of maternal lipid profile, measured in the third trimester, as a predictor of LGA newborns in women with GDM, independent of other known risk factors. Secondarily, we aimed to identify a potential TG cut-off value for increased risk of LGA offspring among women with GDM.

## 2. Materials and Methods

A retrospective and observational study including pregnant women with GDM who gave birth between May 2021 and May 2022 and underwent a lipid profile measurement during the third trimester was performed in a diabetes and pregnancy clinic of a tertiary and academic hospital, Centro Materno-Infantil do Norte, Centro Hospitalar Universitário de Santo António, Portugal. The clinic observes a high volume of pregnant women with GDM, estimated at around 300 per year. Regarding the inclusion and exclusion criteria, both live births and stillbirths were considered. Gestations obtained with human-assisted reproduction techniques were not excluded. Multifetal gestations were excluded.

The diagnosis and classification of GDM was achieved according to the International Association for Diabetes in Pregnancy Study Group (IADPSG) recommendations and the Portuguese National Consensus on Gestational Diabetes: fasting plasma glucose (FPG) ≥ 92 and <126 mg/dL (≥5.1 and <7.0 mmol/L) in the first trimester or 75 g oral glucose tolerance test (OGTT), performed between 24 and 28 weeks of pregnancy, with a FPG ≥ 92 mg/dL (5.1 mmol/L) and/or glucose ≥ 180 mg/dL (10.0 mmol/L) at one hour and/or ≥153 mg/dL (8.5 mmol/L) at two hours [8,9].

Medical nutrition therapy is a first-line treatment in all women diagnosed with GDM, comprising an individually tailored plan with 50–55%, 30%, and 15–20% of the total daily calories deriving from, respectively, carbohydrates, fats, and proteins. Whenever the national standardized glycemic goals (preprandial glucose ≤ 95 mg/dL (≤5.3 mmol/L) and one-hour postprandial glucose ≤ 140 mg/dL (≤7.8 mmol/L)) were not achieved, hypoglycemic drugs (metformin and/or insulin) were associated [9].

Fasting serum glucose, triglycerides, total cholesterol, high-density lipoprotein (HDL), and HbA1c were measured between 30 and 35 weeks of gestation. Serum triglycerides, total cholesterol, and HDL concentration were determined by standard enzymatic methods, using a Cobas Integra 800 analyzer (Roche Diagnostics, Basel, Switzerland). Low-density lipoprotein (LDL) cholesterol concentration was calculated according to the Friedewald’s formula. The samples were collected under fasting conditions.

Relevant demographic data, such as maternal age, obstetric history (parity and previous macrosomia), treatment of GDM, and adverse perinatal events were recorded. Gestational age was determined by ultrasound. Pre-pregnancy body mass index (BMI) was estimated from self-reported height and weight. Percentage of gestational weight gain was calculated by dividing the weight gain by the pre-pregnancy self-reported weight. Hypertriglyceridemia was defined as a TG level greater than the 75th percentile value in our sample, as in the Dathan-Stumpf et al. study [10]. An infant was classified as LGA if its birthweight was above the 90th percentile or small for gestational age (SGA) if below the 10th percentile, based on gestational age and sex-adjusted Portuguese charts [11]. Macrosomia was defined as a birth weight > 4000 g. Prematurity was defined as a birth before 37 completed weeks. Neonatal morbidity was assumed in the presence of at least one of the following: neonatal respiratory distress, neonatal jaundice requiring phototherapy, neonatal hypoglycemia, shoulder dystocia, clavicle fracture, Erb’s palsy, sepsis, or admission to the neonatal intensive care unit.

### Statistical Analysis

Statistical analysis was performed with IBM-SPSS software (Statistical Package for Social Sciences) version 27.0. Distribution normality for continuous variables was verified through histogram observation and kurtosis and skewness analysis. Results are presented as mean ± standard-deviation (*SD*) or median [interquartile range]. Goodness of fit χ^2^-test was used to compare frequencies between categorical variables. Student *t*-test for independent variables was used to compare continuous variables with normal distribution between groups, and the Mann–Whitney test was used in the case of non-normal distribution. A logistic regression model was applied to assess predictors of LGA, adjusting for potential confounders using an enter regression. We grouped age, multiparity, pre-pregnancy BMI, and percentage weight gain. Then we grouped the glycemic control and lipid profile variables with the pre-pregnancy BMI. Results are presented as hazard ratios with 95% confidence intervals. We conducted a receiver operating characteristic (ROC) curve analysis to determine the optimal third-trimester TG cut-off value for predicting LGA newborns. The optimal cut-off point was assessed via searching for the maximum value of sensitivity + specificity − 1 (Youden index). A two-sided *p* value < 0.05 was considered statistically significant.

This study was approved by the local ethics committee of Centro Hospitalar Universitário de Santo António (180-DEFI/188-CE). Patient consent was waived by the ethics committee due to the retrospective nature of the study and full data anonymization.

## 3. Results

A total of 100 singleton pregnant women with GDM and third-trimester lipid profile evaluation gave birth during the study period.

The clinical characteristics of the women and their offspring are presented in Table 1. Ninety percent (*n* = 90) were Portuguese Caucasian women. Mean age was 35.0 ± 5.3 years. Median pre-pregnancy BMI was 25.8 (23.7–30.9) kg/m^2^, and the mean percentage of gestational weight gain was 16.0 ± 10.8%. According to the third-trimester lipid profile, 25.0% (25/100) of the pregnant women had a TG level greater than the 75th percentile value.

Median gestational age at delivery was 39 [38–39] weeks and 8.0% (8/100) were preterm (minimum–maximum 33–41 weeks). Based on the Portuguese charts, 4.0% (*n* = 4) of the newborns were SGA (1 preterm) and 18.0% (18/100) LGA. Seven percent (*n* = 7) were macrosomic, and 35.0% (35/100) had at least one adverse neonatal outcome. No stillbirths or perinatal deaths were recorded.

A comparison of clinical characteristics of pregnant women with and without LGA offspring is shown in Table 2. Pregnant women with LGA offspring presented a higher pre-pregnancy BMI (31.6 (25.6–38.1) kg/m^2^ vs. 25.0 (22.9–29.7) kg/m^2^, *p* = 0.002) and a higher use of metformin (50.0% (9/18) vs. 15.9% (13/82), *p* = 0.004) and insulin therapy (50.0% (9/18) vs. 17.1% (14/82), *p* = 0.005). Adverse neonatal outcomes were also more frequent in this group (61.1% (11/18) vs. 30.8% (24/78), *p* = 0.028). Regarding the third-trimester lipid profile, women with LGA newborns had higher TG levels (296 (187–354) mg/dL vs. 208 (171–253) mg/dL, *p* = 0.027), with no significant differences with respect to LDL and HDL cholesterol. Women with LGA newborns also presented higher third-trimester fasting blood glucose levels (83 (72–92) mg/dL vs. 74 (70–79) mg/dL, *p* = 0.011) and HbA1c levels (5.4 ± 0.4% vs. 5.2 ± 0.4%, *p* = 0.030).

To recognize variables that could predict LGA risk, a logistic regression was performed (Table 3). In the univariate analysis, higher pre-pregnancy BMI, metformin and insulin therapy, third-trimester fasting glucose level, HbA1c, and hypertriglyceridemia were associated with a higher risk of LGA. In the multivariate analysis, only the pre-pregnancy BMI (OR 1.190 (95% CI 1.026–1.380), *p* = 0.022) and hypertriglyceridemia (OR 7.603 (1.695–34.097), *p* = 0.008) were independently associated with LGA newborns, when adjusting for age, multiparity, percentage of gestational weight gain, and glycemic control (HbA1c, fasting glucose and medical therapy).

**Figure 1:**
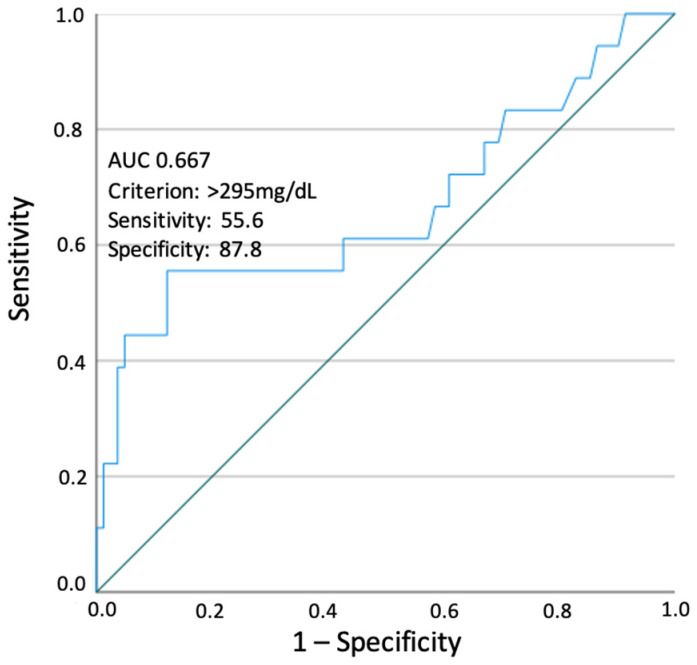
Clinical characteristics of pregnant women with GDM and their offspring.

Using a ROC curve to assess the predictive ability of third-trimester TGs for LGA identification, the optimal TG cut-off value was at 295 mg/dL. Using this cut-off, TGs were a significant predictor of LGA newborns (AUC 0.667 (95% CI 0.503–0.830); *p* = 0.027) with sensitivity and specificity of 55.6% and 87.8%, respectively (Figure 1).

## 4. Discussion

Our findings indicate that pre-pregnancy BMI and the presence of hypertriglyceridemia in the third trimester are independent risk factors for LGA offspring in GDM. Although third-trimester fasting glucose levels and HbA1c were associated with a higher risk of LGA offspring in the univariate analysis, they lost significance as LGA predictors when adjusting for potential confounding variables. This may be due to insufficient power due to sample size, since third-trimester HbA1c was reported as a predictor of LGA offspring in previous studies [12]. The same was observed regarding metformin and insulin use, suggesting an eventual poor glycemic control of these patients.

It is well known that pre-pregnancy obesity, excessive pregnancy weight gain, and poorly controlled GDM increase the risk of LGA newborns and several other neonatal complications, and previous studies have described that insulin resistance is associated with LGA offspring [12,13,14,15,16,17,18]. This well-known relationship might explain why, in our study, we observed a trend for a lower percentage of weight gain during pregnancy in women with LGA newborns, as clinicians are more alert to excessive weight gain in women with higher pre-pregnancy BMI, and therapeutic intervention is more frequent in this group. Moreover, it has been described that maternal insulin resistance causes an increase in plasma TG levels [4]. A positive correlation concerning maternal serum TG levels and birthweight has already been reported [5]. This correlation has been less studied in the GDM population. Several studies have reported that maternal serum TG levels were independently correlated with LGA neonates in women with GDM [6,7,19,20]. However, few studies have considered the glycemic control in the multivariate analysis [6]. In Adank et al.’s study, maternal TG levels were associated with a higher risk of LGA offspring in women with GDM. However, after adjusting for glucose levels, the association weakened and became non-significant [6].

Our findings suggest that third-trimester maternal TG levels can predict LGA newborns independently of age, multiparity, pre-pregnancy BMI, percentage of pregnancy weight gain, and glycemic control (HbA1c, fasting glucose and medical therapy), conferring 6.5 times the odds of having a LGA newborn. Furthermore, we were able to determine a cut-off value of third-trimester serum hypertriglyceridemia (TG > 295 mg/dL) for predicting LGA newborns. Interestingly, our cut-off was similar to that found by Son et al. (295 mg/dL) and marginally different to that found by Jin et al. (313 mg/dL), validating and reinforcing our results [7,21].

As noted above, during pregnancy, several adaptative mechanisms lead to an increase in TG levels, including maternal insulin resistance and elevated estrogen levels, which enhance VLDL production by the liver and decrease its clearance from the circulation [4,22]. Hypertriglyceridemia can be pronounced in GDM pregnancies, and enhanced insulin resistance may explain this relationship [23,24,25]. A recent study pointed to an over-representation of genes associated with placental lipid pathways (compared to those related to glucose metabolism) in GDM pregnancies, with a selective activation of transplacental lipid fluxes [26]. Additionally, higher TG levels lead to an increase in circulating free fatty acids that could act as growth factors and compete for hormones bound to albumin. Thus, there is an increase in free hormone levels that can influence placenta capacity and intrauterine growth and development [27]. All this supports the hypothesis that the accumulation of placental triglycerides represents a regulatory step towards excess fetal adiposity, highlighting the importance of lipids as potential contributors to fetal macrosomia [26,27,28,29].

In our cohort, LGA offspring was linked to a higher prevalence of adverse neonatal outcomes, which is in line with previous studies [12,13,14,15,16,17]. This supports the importance of the early prediction of LGA newborns for guiding the appropriate management and intervention of pregnant women with GDM, namely the timing and methods of delivery.

This study has certain limitations. Due to its retrospective nature, we did not have a lipid profile measurement from all the women diagnosed with GDM in our clinic; thus, a selection bias cannot be completely ruled out, although we consider it unlikely. Moreover, the lipid profile was only measured once, leading to the possibility of inaccuracy, since TG levels are quite variable with diet [30]. However, the therapeutic approach was very uniform in our cohort, with all women receiving a similar medical nutritional plan. Unfortunately, we did not have lipid profile measurements before the third trimester, and we did not have data of offspring growth trajectory during pregnancy. In addition, pre-pregnancy BMI and pregnancy weight gain were calculated from self-reported data; thus, inaccuracy cannot be excluded. However, recent studies indicate a strong correlation between self-reported BMI values and measured BMI values [29,31].

Additionally, in our cohort, women with LGA offspring were quite different, not just in terms of maternal TG levels, but also with respect to pre-pregnancy BMI, fasting glucose levels, and GDM treatment, indicating large derangements of the metabolism. Although fasting glucose levels, HbA1c, and hypoglycemic drug use lost significance in the multivariate analysis, further investigation is needed to confirm if TG levels independently contribute to the incidence of LGA or if they are just a sign of these metabolism derangements. The analysis is constrained by the relatively small sample size of our study, limiting the performance of the ROC curve in determining the optimal TG cut-off value to predict LGA newborns, which is evidenced by the poor sensitivity of the suggested cut-off. Finally, although the incidence of LGA newborns in our study (18.0%) is within the incidence reported in the literature for developed countries, most of our population is Caucasian and from the Mediterranean area, therefore limiting the generalization of our results to other populations [11,32].

## 5. Conclusions

In conclusion, the presence of hypertriglyceridemia in the third trimester of pregnancy was found to be a predictor of LGA offspring among women with GDM, independently of glycemic control, BMI, and gestational weight gain. This highlights the potential role of the lipid profile evaluation during the third trimester. Such evaluation can alert physicians to the risk of LGA offspring and assist in improving management strategies for these pregnancies, since they are associated with more adverse neonatal outcomes. Our findings support the need for further investigations with earlier measurement of serum TG in women with GDM in order to optimize the outcomes of therapeutic interventions and to assure close follow-up. Although maternal serum TG levels do not appear to be a sensitive enough standalone marker of LGA newborns, concurrent first-trimester maternal TG evaluation in pregnancies with a high risk for LGA newborns (including normoglycemic obese women) is needed to confirm the role of TG levels in excessive fetal growth and to validate TG cut-off points for early therapeutic intervention.

## Figures and Tables

**Table 1:** Clinical characteristics of pregnant women with GDM and their offspring.

	*N* = 100
Age (years)	35.0 ± 5.3
Family history of diabetes	27% (27)
Pre-pregnancy weight (kg) *	70 (62–83)
Pre-pregnancy BMI (kg/m^2^) *	25.8 (23.7–30.9)
Multiparity	50.0% (50)
GDM diagnosis	
First trimester	49.0% (49)
Second trimester	51.0% (51)
Metformin therapy	22.0% (22)
Insulin therapy	23.0% (23)
Percentage of gestational weight gain (%)	16.0 ± 10.8
Gestational age at delivery (weeks) *	39 (38–39)
Prematurity	8.0% (8)
Neonatal birth weight (g)	3250.2 ± 496.7
Small for gestational age (SGA)	4.0% (4)
Large for gestational age (LGA)	18.0% (18)
Macrosomia	7.0% (7)
Adverse neonatal outcome ^1^	35.0% (35)
Congenital malformations	3.0% (3)
Third-trimester fasting glucose (mg/dL) *	75 (71–82)
Third-trimester HbA1c (%)	5.2 ± 0.4
Third-trimester TG (mg/dL) *	213 (179–268)
Third-trimester HDL cholesterol (mg/dL)	67.4 ± 13.0
Third-trimester LDL cholesterol (mg/dL) *	147 (117–176)
Hypertriglyceridemia	25.0% (25)

Data are presented as mean ± standard deviation, unless otherwise indicated by * corresponding to data presented as median, with 25th and 75th percentiles. BMI: body mass index; GDM: gestation diabetes mellitus; HDL: high-density lipoprotein; LDL: low-density lipoprotein; LGA: large for gestational age; SGA: small for gestational age; TG: triglycerides. ^1^ At least one adverse neonatal outcome: neonatal respiratory distress, neonatal jaundice (requiring phototherapy), neonatal hypoglycemia, shoulder dystocia, clavicle fracture, Erb’s palsy, sepsis, or admission to the neonatal intensive care unit.

**Table 2:** Comparison of pregnant women with and without large-for-gestational-age offspring.

	Women with LGA Newborns	Women without LGA Newborns	*p*
Age (years)	35.1 ± 5.9	34.9 ± 5.5	0.892
Multiparity	61.1% (11/18)	47.6% (39/82)	0.436
Pre-pregnancy BMI (kg/m^2^) *	31.6 (25.6–38.1)	25.0 (22.9–29.7)	0.002
Percentage of gestational weight gain (%)	14.7 ± 13.1	16.3 ± 10.2	0.579
Metformin therapy ^+^	50.0% (9/18)	15.9% (13/82)	0.004
Insulin therapy ^+^	50.0% (9/18)	17.1% (14/82)	0.005
Adverse neonatal outcome ^1^	61.1% (11/18)	30.8% (24/78)	0.028
Third-trimester fasting glucose (mg/dL) *	83 (72–92)	74 (70–79)	0.011
Third-trimester HbA1c (%)	5.4 ± 0.4	5.2 ± 0.4	0.030
Third-trimester TG (mg/dL) *	296 (187–354)	208 (171–253)	0.027
Third-trimester HDL cholesterol (mg/dL)	64.8 ± 14.4	67.9 ± 12.7	0.362
Third-trimester LDL cholesterol (mg/dL) *	149 (118–154)	146 (115–180)	0.507

Data are presented as mean ± standard deviation, unless otherwise indicated by * corresponding to data presented as median, with 25th and 75th percentiles. ^+^ Six patients were treated with metformin and insulin. BMI: body mass index; HDL: high-density lipoprotein; LDL: low-density lipoprotein; LGA: large for gestational age; TG: triglycerides. ^1^ At least one adverse neonatal outcome: neonatal respiratory distress, neonatal jaundice (requiring phototherapy), neonatal hypoglycemia, shoulder dystocia, clavicle fracture, Erb’s palsy, sepsis, or admission to the neonatal intensive care unit.

**Table 3:** Predictors of large-for-gestational-age newborns.

	Univariate Analysis		Multivariate Analysis	
	Crude OR (CI 95%)	*p*	Adjusted OR (CI 95%)	*p*
Age (years)	1.007 (0.917–1.105)	0.891	0.964 (0.856–1.085)	0.540
Multiparity	1.733 (0.611–4.912)	0.301	1.139 (0.283–4.578)	0.855
Pre-pregnancy BMI (kg/m^2^)	1.127 (1.044–1.215)	0.002	1.190 (1.026–1.380)	0.022
Percentage of gestational weight gain (%)	0.986 (0.939–1.036)	0.575	1.054 (0.970–1.145)	0.213
Metformin therapy	5.308 (1.771–15.908)	0.003	4.209 (0.856–20.058)	0.077
Insulin therapy	4.857 (1.636–14.423)	0.004	0.790 (0.121–5.138)	0.805
Third-trimester fasting glucose (mg/dL)	1.077 (1.025–1.132)	0.003	1.073 (0.999–1.150)	0.054
Third-trimester HbA1c (%)	4.042 (1.107–14.759)	0.035	1.153 (0.197–6.741)	0.874
Hypertriglyceridemia	5.583 (1.886–16.528)	0.002	7.603 (1.695–34.097)	0.008

BMI: body mass index.

## Data Availability

The data that support the findings of this study are available from the corresponding author, upon reasonable request. The data are not publicly available due to legal and ethical reasons.
